# Factors influencing mobility in community-dwelling older adults during the early COVID-19 pandemic: a cross-sectional study

**DOI:** 10.1186/s12889-023-16553-3

**Published:** 2023-08-28

**Authors:** Hyori Kim, Juah Kim, Jiyeon Ha

**Affiliations:** 1 College of Nursing, Seoul National University, Seoul, Republic of Korea; 2 Department of Nursing, Korean Bible University, Seoul, Republic of Korea; 3 College of Nursing, Research Institute of Nursing Science, Ajou University, 164 World cup-ro, Yeongtong-gu, Suwon-si, 16499 Gyeonggi-do Republic of Korea

**Keywords:** Aged, COVID-19, Depression, Mobility limitation, Sedentary behavior

## Abstract

**Background:**

In older adults, mobility is important for maintaining their independence and quality of life, and it influences their physical, cognitive, and social health. This study aimed to identify the physical and psychosocial factors that affected the mobility of community-dwelling older adults, aged 65 years or older, who were socially isolated during the coronavirus disease 2019 (COVID-19) pandemic due to stay-at-home policies.

**Methods:**

The participants in this study were 214 community-dwelling older adults in Korea, and a cross-sectional survey was conducted from December 2020 to January 2021. Variables included participants’ general characteristics, mobility, sitting time, depression, social support, and cognitive function.

**Results:**

Multiple linear regression analysis showed that the factors influencing older adults’ mobility during the COVID-19 pandemic were depression (β=-0.29, *p* < .001), age (65–74 years old) (β = 0.19, *p* = .002), a lower level of education (β=-0.17, *p* = .006), two or more comorbidities (β=-0.18, *p* = .001), sitting time (β=-0.17, *p* = .004), and the ability to drive a vehicle (β = 0.14, *p* = .017).

**Conclusions:**

Home healthcare interventions are needed to limit psychosocial issues and improve mobility for older adults who had limited mobility during the COVID-19 pandemic.

## Background

Physical and social health problems affecting the older population are drawing attention as population aging becomes significant on a global scale. In older adults, mobility s an important predictor of successful aging [1] and is recognized as a significant concept for resolving health problems [2]. Mobility has been reported to influence the admission rate, fall, morbidity and mortality rate [3, 4], and it is closely related to the quality of individuals’ physical and psychological experiences. Coronavirus disease 2019 (COVID-19), which has spread worldwide since 2020, has presented a major threat to the lives and health of older adults. Older adults with underlying diseases or frailty are more vulnerable to COVID-19 infection [5]. Therefore, many governments recommended staying at home and maintaining distance from neighbors [6, 7]. Such measures added physical constraints to the daily lives of older adults; reducing their mobility and causing negative effects on their health, such as cardiovascular diseases and autoimmune disorders [8]. In addition, decreased cognitive stimulation due to social isolation can worsen the symptoms of dementia [9], and social isolation and loneliness can impair the health of older adults [10].

Mobility is a concept that includes ambulation, moving from a bed to a chair, walking or engaging in activities in leisure and everyday life, exercising, driving, and using a variety of public transit modes [11, 12]. Although mobility in older adults has been defined differently according to the context, it refers to the ability to move to carry out basic functions independently without needing help [13]. In a global report on aging and health, the World Health Organization (WHO) defined mobility in older adults as a person’s physical ability to move from one place to another [14], and Webber et al. defined mobility as the ability to move from one’s home to an environment beyond one’s neighborhood and community, whether independently or using an assistive means of transportation [2].

Webber et al., who published a comprehensive framework regarding mobility of older adults, identified that mobility affected physical, psychosocial, cognitive, environmental, and economic predictors and sex, cultural, and personal life [2]. Meyer et al. later reported that physical, cognitive, psychosocial, and environmental determinants were factors affecting mobility, reporting results similar to the study of Webber et al. [15]. In a study of older people living in communities without mobility restrictions, physical and psychological measures were found to be significant factors related to real-life mobility, but cognitive and social measures were not influencing factors [16]. However, other previous research has reported that mild to moderate cognitive impairment in older persons living in the community was a potential determinant of mobility [17]. According to a recent study, driving, social support, and walking speed were the factors that had the most influence on life-space mobility [18]. The factors known to affect mobility in older adults include variables closely related to depression, geographical location, safety, falls, participation in physical activity, sex, ethnicity, level of education, and marital status [12, 15], muscular strength and balance disorders [19], chronic diseases [20], sedentary time [21], and physical function [22]. In summary, physical, psychological, cognitive, and environmental factors, as well as demographic characteristics, were identified as factors that commonly affect mobility in older adults.

This study attempted to investigate changes in the daily lifestyle of older people during the COVID-19 pandemic, a period where social welfare centers were shut down and it was recommended to maintain distance from one’s neighbors and stay at home [23]. Therefore, this study aimed to investigate factors amid the COVID-19 pandemic, which influenced mobility in community-dwelling older adults, aged 65 years or older, including multidimensional predictors such as physical, psychological, cognitive, and social support, as well as general characteristics.

## Methods

### Participants and procedures

This cross-sectional study investigated the mobility, physical, and psychosocial characteristics of older adults and aimed to identify factors that influenced their mobility. The participants in this study were community-dwelling older adults, aged 65 years or over, who had the ability to communicate and resided in a metropolitan city. G*Power version 3.1.9.7 was used to determine the number of participants. Assuming a multiple regression analysis with a significance level of 0.05, an effect size of 0.15, a test power of 0.95, 14 predictors, and a dropout rate of 10%, 214 was determined as the number of participants to be recruited. This study was approved (KYU-2020-098-01) by the institutional review board of the university where the researcher works. The 214 participants were surveyed from December 29, 2020 to January 19, 2021, and their data were analyzed in full without dropouts.

### Materials

Participants’ sex, age, level of education, marital status, employment, monthly income, number of chronic diseases, and ability to drive a vehicle were measured as the general characteristics.

The Physical Functioning Scale (PFS) for Community-Dwelling Older Persons, developed by Lee et al., was used to measure mobility in participating older adults [24]. The scale uses a 4-point Likert scale and consists of 10 questions total, 5 questions about mobility and 5 questions about self-care. The 5 questions about mobility, were used in this study, and a higher score indicated greater mobility. The reliability of the 5 mobility questions was 0.89 when the scale was developed, and Cronbach’s ⍺ for the reliability of the 5 mobility questions in this study was 0.91.

To measure sitting time, this study used a question from the International Physical Activity Questionnaire (IPAQ) developed by WHO about how much time was spent sitting during the last 7 days [25, 26]. Time spent sitting includes time spent at work, at home, while doing coursework, during leisure time, at a desk, visiting friends, reading, or reclining to watch television.

To measure participants’ mental health, this study used the short form of the Geriatric Depression Scale (SGDS-K), which is Kee’s Korean revision of the Geriatric Depression Scale Short Form [27], developed by Sheik and Yesavage [28]. This survey consists of a total of 15 questions, and each question is given 0 points for “no” and 1 point for “yes.” The negative questions are reverse-calculated for a total range of 0–15 points, with higher scores correlated to higher levels of depression. Cronbach’s ⍺ was 0.88 for reliability in Kee’s study [27], and Cronbach’s ⍺ in this study was 0.85.

Social support was measured using the ENRICHD Social Support Instrument (ESSI) developed by ENRICHD (Enhancing Recovery in Coronary Heart Disease) [29] and used widely in international studies of community-dwelling older adults. The Korean version of the instrument translated by Jeon et al. was used [30]. This tool consists of 6 items, and the total score is calculated by giving 1 point for “yes” and 0 points for “no” to the emotional, informational, and instrumental support questions. Higher scores indicate a higher level of social support. Cronbach’s ⍺ was 0.93 when the instrument was developed, 0.84 in the study by Jeon et al. [30], and 0.90 in this study.

As an instrument to evaluate participants’ cognitive function, the Korean version of the Mini-Mental State Examination-Dementia Screening (MMSE-DS), developed by Kim et al. [31], was used. Since its development by Folstein et al. [32], the MMSE has been used globally to assess the level of cognitive function disorder quantitatively and monitor changes in cognitive function based on iterative measurements. The tool consists of 19 questions and is scored 0 to 30: 10 points for orientation, 6 points for memory, 5 points for attention, 3 points for language, 3 points for registration, 1 point for copying figures, and 2 points for judgment and common sense. The total score is calculated according to sex, level of education, and age in accordance with the guideline for the use of the measure. A score less than the standard MMSE-DS test score means cognitive decline, and a score higher than the standard score means a normal result. A higher score reflects higher levels of cognitive function. Cronbach’s ⍺ for the Korean version of the MMSE-DS was 0.83 [31], and Cronbach’s ⍺ for this study was 0.79.

### Statistical analysis

The data collected were analyzed at a significance level of *p* < .05 using SPSS version 26.0 (IBM Corp., Armonk, NY, USA). The general characteristics, mobility, sitting time, depression, social support, and cognitive function variables of the participants were analyzed with descriptive statistics such as frequency, percentage, mean, and standard deviation.

Differences in the mobility score were analyzed using the independent t-test and one-way analysis of variance, and the post-hoc analysis was conducted using the Scheffé test. In order to test the normality of the data, the Shapiro-Wilk test was performed when the number (n) of one subgroup was less than 30, and the non-parametric test was performed for data that did not have a normal distribution. The Mann-Whitney U test and Kruskal-Wallis test were used for the analysis if the data did not show a normal distribution, and the post-hoc analysis was conducted using the Dunn-Bonferroni test. Correlations among mobility, sitting time, depression, social support, and cognitive function were analyzed using Pearson’s correlation. Stepwise multiple linear regression was conducted to investigate the factors associated with mobility in older adults, with a focus on the variables found to be significant in the univariate analysis.

In the multiple regression model, the most suitable regression model can be selected by sequentially selecting or removing the variables to be used one by one. Stepwise regression analysis is a method of re-examining the importance of already selected variables to eliminate these disadvantages by removing low-importance variables [33]. Therefore, a stepwise selection method was applied to construct the most suitable multiple regression model after determining the optimal variable combination.

## Results

### Differences in mobility according to participants’ general characteristics

Of the 214 participants, there were 94 (43.9%) male participants and 120 (56.1%) female participants (Table 1). The mean age was 74.75 ± 6.01 years, and there were 103 (48.1%) older adults aged 65–74 years, who accounted for the majority. As for the level of education, 84 (39.3%) participants had received an elementary or lower level of education, 96 (44.9%) participants had received a middle or high school education, and 34 participants had received a college or higher level of education. Of the participants, 134 (62.6%) were married, and 65 (69.6%) had a job. There were 155 (72.4%) participants with a monthly income of 800 USD or less and 115 (53.7%) participants with 2 or more chronic diseases. Sixty-seven participants (31.3%) answered that they drive.

Mobility showed significant differences in sex (t = 3.20, *p* = .002) and age (*p* < .001). According to the post-hoc analysis, mobility was significantly greater among the male participants than among the female participants and in the group aged 65–74 years than in the groups aged 75–84 years and 85 years or older. The low-education group (elementary school or lower) had significantly lower mobility than the other two education groups (F = 17.82, *p* < .001). Mobility showed significant differences according to marital status (t = 2.57, *p* = .011), employment (t = 4.86, *p* < .001), monthly income (*p* = .014), number of chronic diseases (*p* < .001), and whether the participant drives a vehicle (t = 5.20, *p* < .001). The post-hoc analysis showed that mobility was significantly lower among single participants and employed participants. The group with a monthly income of 800 USD or less and those who had two or more chronic diseases had significantly lower mobility than the other groups. The participants who did not drive a vehicle had significantly lower mobility than those who did (Table 1).

**Table 1:** Differences in mobility by general characteristics (*N*=214)

Variables	Category	n (%) or M±SD	Mobility	t or F	***p***
			M±SD		
Sex	Male	94 (43.9)	12.96±3.01	3.20	.002^**†^
	Female	120 (56.1)	11.63±3.01		
Age (years)	65-74^a^	103 (48.1)	13.32±2.26	-	<.001^**∥^ a>b,c^¶^
	75-84^b^	95 (44.4)	11.14±3.41		
	≥ 85^c^	16 (7.5)	11.50±3.18		
		74.75±6.01			
Education	≤ Elementary school ^a^	84 (39.3)	10.88±3.32	17.82	<.001^**‡^ a<b,c^§^
	Middle/high school ^b^	96 (44.9)	12.73±2.74		
	College or above ^c^	34 (15.9)	14.06±1.67		
Marital status	Married	134 (62.6)	12.63±3.08	2.57	.011^*†^
	Single	80 (37.4)	11.53±2.96		
Employment	Yes	65 (69.6)	13.49±2.17	4.86	<.001^**†^
	No	149 (30.4)	11.66±3.24		
Monthly income (currency: USD)	0-800 ^a^	155 (72.4)	11.84±3.17	-	.014*^∥^ a<b^¶^
	801-2400 ^b^	45 (21.0)	13.27±2.60		
	≥ 2401^c^	14 (6.5)	13.00±2.51		
Number of chronic diseases	0 ^a^	29 (13.6)	13.59±2.29	-	<.001^**∥^ a,b>c^¶^
	1 ^b^	70 (32.7)	12.97±2.70		
	≥ 2^c^	115 (53.7)	11.41±3.23		
		1.81±1.23			
Ability to drive a vehicle	Yes	67 (31.3)	13.61±2.40	5.20	<.001^**†^
	No	147 (68.7)	11.58±3.14		

^*^P-value<0.05, ^**^P-value<0.01

^†^Independent T-test; ^‡^Analysis of variance(ANOVA); ^§^Scheffé test; ^∥^Kruskal-Wallis test; ^¶^Mann-Whitney U test with Dunn-Bonferroni correction

### Correlations among mobility, sitting time, depression, and cognitive function

The mean mobility score of the participants was 12.22 ± 3.07 points, and the mean sitting time was 6.27 ± 2.13 h/day. The mean depression score was 3.78 ± 3.39 points, and the mean social support score was 5.32 ± 1.56 points. The mean cognitive function score was 24.75 ± 3.95 points (Table 2).

**Table 2:** Descriptive statistics of variables

Variables	M ± SD	Range
Mobility^a^	12.22 ± 3.07	0–15
Sitting time (hours/day)	6.27 ± 2.13	0–24
Depression^b^	3.78 ± 3.39	0–15
Social support^c^	5.32 ± 1.56	0–6
Cognitive function^d^	24.75 ± 3.95	0–30

^a^ Mobility measured by PFS (Points)

^b^ Depression measured by SGDS-K (Points)

^c^ Social support measured by ESSI (Points)

^d^ Cognitive function measured by MMSE-DS (Points)

For mobility, depression (*r*=-.42, *p* < .001) and sitting time (*r*=-.37, *p* < .001) exhibited a statistically significant negative correlation. On the other hand, social support (*r* = .16, *p* = .018) and cognitive function (*r* = .32, *p* < .001) showed a statistically significant positive correlation (Table 3).

**Table 3:** Correlations among variables

Variables	1	2	3	4	5
	r (*p*)	r (*p*)	r (*p*)	r (*p*)	r (*p*)
1. Mobility^a^	1				
2. Sitting time (hours/day)	− 0.37^**^	1			
	< 0.001				
3. Depression^b^	− 0.42^*﻿*^	0.34^**^	1		
	< 0.001	< 0.001			
4. Social support^c^	0.16^*^	0.034	− 0.30^**^	1	
	0.018	0.620	0.001		
5. Cognitive function^d^	0.32^*^	− 0.21^**^	− 0.23^**^	0.16^*^	1
	0.013	0.002	0.001	0.018	

^*^P-value < 0.05, ^**^ *P*-value < 0.01

^a^ Mobility measured by PFS (Points)

^b^ Depression measured by SGDS-K (Points)

^c^ Social support measured by ESSI (Points)

^d^ Cognitive function measured by MMSE-DS (Points)

### Factors influencing participants’ mobility

Stepwise multiple linear regression was conducted to identify the factors influencing participants’ mobility. For the independent variables, the general characteristics (sex, age, level of education, marital status, employment, monthly income, number of chronic diseases, and ability to drive a vehicle), physical and psychosocial variables (sitting time, depression, and social support), and cognitive function were inserted by stages. The categorical variables of age, level of education, monthly income and number of chronic diseases were treated as dummy variables in the analysis. The multiple regression analysis showed that the tolerance was 0.81–0.94, which was higher than 0.1, and since the variance inflation factor (VIF) ranged from 1.06 to 1.24, which did not exceed 10, the multicollinearity problem between the independent variables was ruled out. For the test for independence of residuals before the analysis, since the Durbin-Watson statistic was 2.23, which was close to 2, there was found to be no autocorrelation, and the regression model was found to be significant (F = 22.89, *p* < .001).

The most significant influence on participants’ mobility was depression (β=-0.29, *p* < .001), followed by being aged 65–74 years compared to the group aged 85 years or older (β = 0.19, *p* = .002), being an elementary graduate compared to the group that received a college or higher level of education (β=-0.17, *p* = .006), having two or more chronic diseases compared to participants without chronic diseases (β=-0.18, *p* = .001), sitting time (β=-0.17, *p* = .004), and being able to drive a vehicle (β = 0.14, *p* = .017). Reduced mobility was associated with more intense depression, a longer sitting time, a lower level of education (elementary school or lower), and multiple chronic diseases. Furthermore, greater mobility was correlated to being aged 65–74 years and being able to drive a vehicle. The total explanatory power for the models was 38.0% (Table 4).

**Table 4:** Factors influencing mobility

Variables	B	SE	β	t (*p*)
(Constant)	14.88	0.61		24.34 (< 0.001)
Depression^a^	-0.26	0.05	− 0.29	-4.93 (< 0.001)
Age (years) 65–74 (ref.= ≥ 85)	1.13	0.36	0.19	3.19 (0.002)
Education ≤ Elementary school (ref.= college or above)	-1.04	0.38	− 0.17	-2.75 (0.006)
Number of chronic diseases ≥ 2 (ref.= none)	-1.12	0.34	− 0.18	-3.27 (0.001)
Sitting time (hours/day)	-0.24	0.08	− 0.17	-2.87 (0.004)
Ability to drive a vehicle (ref.= No)	0.93	0.39	0.14	2.41 (0.017)
R^2^ = 0.40, Adj.R^2^ = 0.38, F = 22.89, *p* < .001				

*B *Unstandardized estimates, *SE *Standard error, *β *Standardized estimates, *Adj. R*^2^Adjusted R^2^

^a^Depression measured by SGDS-K (Points)

## Discussion

This study was conducted to identify the factors influencing the physical and psychosocial health characteristics of older adults on their mobility during the COVID-19 pandemic. In this study, the mean mobility score was 12.22 ± 3.07, corresponding to 73.32 points out of 100. This result was slightly lower than the score of 75.8 for older people aged 65 or older at the time of development of this research tool [24]. Decreased physical function or weakness that goes along with aging causes limitations in daily life, reduces physical activity or mobility, and can ultimately have a negative impact on the health of older adults [34]. The concept of mobility has recently been expanded to the concept of life-space mobility, which is not simply walking ability—instead, it refers to moving from one place to another destination, and is defined as a spectrum of geographical areas that extends from one’s residence to a distant destination [34, 35]. Maintaining the level of life-space mobility even after retirement is an important factor for successful aging in terms of preventing shrinkage of social networks and social isolation [36]. In addition, there are previous research results that the mobility of older adults ultimately has a positive effect on the life satisfaction of older people [37–39]. Therefore, it is necessary to develop various supportive policies and programs that can improve mobility by monitoring the factors that affect the mobility of older adults. In addition, given the physical limitations of older adults, it is necessary to consider building a transportation environment or infrastructure related to social participation in an age-friendly manner.

An analysis of the factors influencing the mobility of older adults in this study showed that the regression model including depression, age, level of education, the number of chronic diseases, sitting time, and the participant’s ability to drive a vehicle was significant and exhibited an explanatory power of 38.0%. Of those, the largest factor influencing mobility in older adults was depression. A cross-sectional study of a community cohort of older adults aged 70 or older also showed that high levels of depressive symptoms were related to gait functions such as velocity, stride, and swing time variability [40]. Depression is a common mental disorder characterized by sadness, loss of interest or pleasure, feelings of guilt or low self-worth, disturbed sleep or appetite, feelings of tiredness, and poor concentration. It impacts the maintenance of daily life functions and independence and is recognized as the single largest contributor to global disability increase [41]. In particular, because the COVID-19 pandemic continued for more than a year, unprecedentedly strong quarantine guidelines for reducing the spread of COVID-19 in communities increased restrictions on the daily lives of older adults [42]. During this period, depression emerged as such an important health problem in Korea that “corona blue,” a word made by combining “coronavirus” and “blue,” meaning “depression,” was recognized as a significant social problem [43]. Moreover, since the study data were collected while social distancing and home quarantine policies actively restricted social events, older adults experienced negative emotions such as lethargy, loneliness, anxiety, and depression as they stayed at home alone for longer periods of time [5, 23, 44]. In addition, since older adults were able to consume information about COVID-19 from a variety of sources, including news outlets, online media, and emergency text messages, having excessive information about the pandemic may have increased their anxiety and stress, potentially intensifying their depression [23]. Based on prior studies showing that people who have experienced depression are likely to have a significant decrease in physical activity [45] or muscular strength, or to be in frail health [46], it can be inferred that the fear, stress, and depression caused by the COVID-19 pandemic resulted in the health decline of older adults, which in turn caused their decreased mobility [47]. Therefore, various policies and support programs that reduce psychosocial problems due to the spread of new infectious diseases should be developed and prepared in advance. The development of home healthcare systems that can increase older adults’ mobility at home also needs attention.

This study also showed that sitting time influenced mobility in older adults. According to prior studies, as older adults spend more time sitting, the incidence of cardiovascular diseases increases [48], quality of life declines [49], and their risk of mortality rises [50]. In this study, the mean time that participants spent sitting during the COVID-19 pandemic was 6.27 h a day. Although a study suggested that the threshold causing negative health outcomes in older adults is 7 h a day [51], another study conducted among older adults in Spain showed that sitting for more than 4 h a day was correlated to a reduced physical fitness level [52]. According to a large-scale study conducted in six countries, if one spends more than 4 h a day sitting, there may be an association with decreased physical activity and poorer subjective health conditions [53]. In particular, the increased time that older adults spent sitting was attributed to spending more time at home while stay-at-home and strict social distancing policies were implemented due to the COVID-19 pandemic [23]. However, since some sedentary behaviors (e.g., using a computer during one’s leisure time) are known to be correlated with a reduced risk of dementia [54], additional studies should focus on outcomes in relation to both sitting time and sedentary behaviors associated with social and cognitive activities.

In this study, social support and cognitive function were positively correlated with mobility. This supports the results of previous studies, in which social support had a positive effect on mobility in older adults [55]. Mobility was also more limited in older people who had diminished cognitive function [56]. However, these two variables were not significant in the results of the stepwise regression analysis, so they were excluded from the final model. This may reflect the fact that most of the participants did not have problems with cognitive function and the overall weakening of social support during the COVID-19 pandemic [57]. It was reported that COVID-19 aggravated cognitive decline in older adults [58], since the social isolation caused by COVID-19 can have a negative impact on reduced mobility, particularly in older persons compared to other age groups [59], After the COVID-19 pandemic, additional research is needed to determine whether social support and cognitive function affect mobility in older adults.

Of the general characteristics in this study, age, level of education, the number of chronic diseases, and the ability to drive a vehicle were the factors that influenced older adults’ mobility. The result that the youngest group of participants (aged 65–74 years) had greater mobility than the oldest group (aged 85 years or older) corresponds to the study by Picazzo-Palencia [60], which found that the older an individual becomes, the more their basic and intermediate mobility decreases. Since it is likely that aging itself may cause physical function to deteriorate and mobility to be increasingly limited, focused health management becomes proportionally more important for improving mobility in the oldest group. In addition, the current study’s result regarding the influence of a low level of education is similar to the results of prior studies [61, 62]. Low socioeconomic status and restricted life-space mobility are often known to coexist with obesity, reduced cognition, and poorer physical performance [62]. Since prior studies showed that higher levels of education were associated with being more informed about COVID-19 [63], it is possible that participants with a high level of education had stronger decision-making skills about their range of physical activity relative to their knowledge of COVID-19 and its risks. Health programs supporting the ability of older adults with a low level of education to understand, interpret, and use information about health should be developed and provided to communities.

In addition, having two or more chronic diseases influenced mobility. This result corresponds to prior studies’ findings that the prevalence and number of comorbidities limited activities of daily living and independence [64, 65]. Since comorbidities were found to influence the mobility of older adults in this study, we believe that health management systems that closely monitor individuals with multiple chronic diseases are necessary to improve the mobility and independence of community-dwelling older adults. Moreover, prior studies have reported that older adults engaged in physical activity more actively if they could drive a vehicle [19, 66]. Driving a vehicle can increase older adults’ mobility by enabling access to outdoor activities and may facilitate the expansion of their social networks [38]. Although there have been active discussions on aging-related risks for older drivers, in light of the finding of a previous study that the loss of driving had a negative impact on well-being [67], it may be beneficial to maintain older adults’ ability to drive independently as long as their physical function allows.

This study has several limitations. First, since this study was conducted in older adults residing in a metropolitan city, it is difficult to generalize the results to older adults in other residential environments, such as rural areas and small cities. Second, because the data were collected when strong social distancing and home quarantine policies were active in South Korea, the results of this study should be applied in consideration of that distinctive context. Third, sociocultural differences between the participants in this study and older adults in other cultures should be considered when applying the results of this study to older adults in other cultures.

Since this study was conducted while social distancing was active due to the COVID-19 pandemic, it has the advantage of providing useful information to identify general and psychosocial characteristics influencing older adults’ mobility during the prevalence of new infectious diseases. In addition to factors known already to affect older adults’ mobility, such as depression, sitting time, age, and the number of chronic diseases, constraints related to COVID-19 have highlighted two other factors in this study: level of education, which affected the ability to obtain and interpret knowledge about COVID-19, and the ability to drive a vehicle, which affected access to social events.

## Conclusions

This study aimed to determine the general and psychosocial characteristics that may affect older adults’ mobility during the prevalence of new infectious diseases by examining the factors that influenced the mobility of community-dwelling older adults aged 65 years or older during the early COVID-19 pandemic. The study found that the influential factors included depression, age, level of education, the number of chronic diseases, sitting time, and ability to drive a vehicle. Mobility plays a substantial role in the health of older adults because improving the mobility of older adults is a prerequisite for successful aging by maintaining physical function and engagement with life. It is necessary to prepare various policy measures and programs and expand resources to strengthen the mobility of older adults. In particular, older adults who had limited mobility during the COVID-19 pandemic should receive home healthcare interventions that can limit psychosocial issues and improve mobility. In addition, during the spread of new infectious diseases, early intervention would be necessary for older adults with factors negatively influencing their mobility. We also suggest that follow-up studies compare effects on older adults’ mobility before, during, and after the COVID-19 pandemic.

## Data Availability

The datasets used and/or analyzed during the current study are available from the first author upon reasonable request.
